# Weight Gain Among Cancer Patients Receiving Chemotherapy—Facts and Numbers

**DOI:** 10.1002/jcsm.13694

**Published:** 2025-02-19

**Authors:** Muhammad Shahzeb Khan, Javed Butler, Markus Anker

**Affiliations:** ^1^ Department of Medicine Baylor College of Medicine Temple Texas USA; ^2^ Division of Cardiology The Heart Hospital Plano Plano Texas USA; ^3^ Baylor Scott and White Research Institute Dallas Texas USA; ^4^ Department of Medicine University of Mississippi Medical Center Jackson Michigan USA; ^5^ Department of Cardiology, Angiology and Intensive Care Medicine CBF Deutsches Herzzentrum der Charité Berlin Germany; ^6^ Corporate Member of Freie Universität Berlin and Humboldt‐Universität zu Berlin Charité – University Medicine Berlin Berlin Germany; ^7^ Partner Site Berlin German Centre for Cardiovascular Research (DZHK) Berlin Germany; ^8^ Berlin Institute of Health Center for Regenerative Therapies (BCRT) Berlin Germany

**Keywords:** cancer patients, chemotherapy, facts and numbers, weight gain

## Abstract

Cachexia affects up to 60% of patients with lung cancer, with its prevalence rising up to 80% in advanced stages of disease. In approximately 20% of cases, it is the primary cause of mortality. Five studies, including a total of 4467 patients, across range of cancer types reported data on weight gain in cancer patients undergoing chemotherapy. Across all five studies, an average of 18.3% of patients experienced weight gain > 5% (816 out of 4467 patients). The frequency of weight gain > 5% was highest among breast cancer patients, 18.9% in Pedersini et al (*n* = 169) and 33.0% in Sella et al (*n* = 687). In NSCLC patients, weight gain was reported in 18.3% in patients in Patel et al (*n* = 2301) and 11.7% in Roeland et al (*n* = 1030). In contrast, colorectal cancer patients showed only 5.7% of weight gain > 5% (Zutphen et al, *n* = 280). Additionally, weight loss > 5% was reported in 15.1% of breast cancer patients and 28.3% of colorectal cancer patients. Despite weight loss being quantified as a common endpoint in clinical trials focused on cancer cachexia, there is limited data on the impact of weight gain as a marker of a positive outcome among cancer patients. Studies have shown that weight gain of more than 5% within 3 months in NSCLC patients can be associated with improvement in overall survival (OS) and progression‐free survival (PFS) scores. In this post hoc analysis by Roeland et al., the authors defined different percentage cut‐off values for maximum weight gain among patients with non–small cell lung cancer within 3 months of starting platinum‐based chemotherapy. Among all categories, namely, weight gain > 0%, > 2.5% and > 5%, a significant benefit in overall and progression‐free survival was seen and was comparable among all groups. These findings highlight the clinical significance of incorporating strategies that encourage weight gain and to prevent weight loss at the least among cancer patients. Along with further delving into the prognostic value of weight gain and developing methods to encourage this response among cancer patients, future studies should use standardized assessment tools to identify weight gain that could be attributed to underlying pathologic processes such as oedema and congestion. We also suggest that monitoring and reporting of weight changes should be done in all cancer trials.

Cancer cachexia is a multifactorial syndrome with a complex pathophysiology and significantly impacts patient quality of life, response to therapy and survival. Cachexia is observed in approximately 80% of patients with upper gastrointestinal malignancies and 60% of those with lung cancer [1]. The timely identification of cancer cachexia is imperative. Using Fearson et al.'s criteria, cancer cachexia can be diagnosed based on changes in weight, such as weight loss of > 5% in 6 months, or > 2% with a body mass index (BMI) of < 20 kg/m^2^, or the presence of sarcopenia [2]. Alternatively, it can also be diagnosed in the absence of weight changes, using BMI < 20 kg/m^2^ along with three out of five factors, namely, abnormal biochemistry, fatigue, anorexia, reduced muscle strength and depleted fat‐free mass [3].

Several treatment modalities are currently under investigation for cachexia. Although megosterol acetate has resulted in weight gain by stimulating increased appetite, it has not positively impacted quality of life [4]. Similarly, anamorelin, a ghrelin receptor agonist, demonstrated a beneficial effect on lean and fat body mass but not on hand grip strength; thus, it has not received regulatory approval in Europe and the United States [5]. A selective androgen receptor modulator, enobosarm, showed a significant change in lean body mass and stair‐climb power when analysed using guidelines outlined by the European Medicines Agency (EMA). However, the changes were no longer significant when analysed according to the US Food and Drug Administration (FDA). Owing to differences in clinical trial design, inclusion criterion and definition of endpoints, a consensus regarding appropriate treatment modalities for cachexia has yet to be reached [6]. Additionally, the role of nutritional modifications along with physical exercise regimens must be incorporated into the healthcare plan for cachexia patients. This is currently being explored and implemented among heart failure patients with cardiac cachexia, but further identification of a multimodal approach to management is required in other forms of cachexia [4].

A major portion of cancer patients suffer from cachexia, with approximately 16.5 subjects per 10 000 people of the population of the United States, and 15.8 subjects per 10 000 people of the population of Europe, contributing to an estimated 30% of deaths among them. The prevalence of cachexia rises from 50% to over 80% in the terminal phase of illness, and it is the primary cause of mortality in approximately 20% of the cases. [1] Identifying cancer cachexia as an orphan disease, which is characterized as a rare disorder that receives special clinical development, can fill the gap in effective therapeutic modalities. Cancer cachexia due to individual cancers, namely, gastric and lung cancer in the United States and melanoma and colorectal cancer in the EU, meets the criteria for orphan disease [7]. Currently, weight loss is commonly used as a prognostic factor among cancer patients, often signalling poorer outcomes. Sarcopenia, the loss of muscle mass and strength along with adipose tissue depletion, is considered major hallmarks of cancer cachexia, and studies identifying them as poor prognostic factors have shown results of potential clinical significance [8, 9]. In a novel concept, Lena et al. demonstrated low left ventricular mass among advanced cancer patients with cachexia, with possible implications on cardiac function possibly resulting in arrhythmias and heart failure. This phenomenon was identified as cardiac‐wasting–associated cardiomyopathy and offered potential use as a prognostic factor among cancer patients, as it led to decreased functional status and survival [10]. Surprisingly, despite weight loss being quantified as a common endpoint in clinical trials focused on cancer cachexia, there are limited data on the impact of weight gain as a marker of a positive outcome among cancer patients.

Five studies, including a total of 4467 patients, reporting data on percentage gain in weight among cancer patients undergoing chemotherapy were identified (Table 1) [11, 12, 13, 14, 15]. These studies included various cancer types, including breast, colorectal, and non–small cell lung cancer (NSCLC). The timing and inclusion criteria for assessing weight changes varied across studies. Across all five studies, an average of 18.3% of patients experienced weight gain > 5% (816 out of 4467 patients). The frequency of weight gain > 5% was highest among breast cancer patients, 18.9% in Pedersini et al and 33.0% in Sella et al. In NSCLC patients, weight gain was reported in 18.3% in patients in Patel et al. and 11.7% in Roeland et al. In contrast, only 5.7% patients with colorectal cancer showed weight gain > 5%. Weight loss was reported in varying frequencies across studies. For example, 15.1% of breast cancer patients experienced weight loss > 5%, whereas colorectal cancer patients exhibited a rate of 28.3%. Studies show that cancer patients who experience modest increase in weight (> 5%) during the first 3 months of adjuvant chemotherapy can show an improvement in overall survival (OS) and progression‐free survival (PFS), including both locoregional and distant metastasis‐free survival. These findings underscore the importance of strategies to promote weight gain and prevent weight loss in patients with cancer [14, 16].

**TABLE 1:** Summary of studies demonstrating percentage increase in weight among cancer patients undergoing chemotherapy.

Parameters	Pedersini et al. [11] (*n* = 169)	Zutphen et al. [12] (*n* = 280)	Sella et al. [13] (*n* = 687)	Patel et al. [14] (*n* = 2301)	Roeland et al. [15] (*n* = 1030)
Date published	2023 September	2019 November	2022 July	2016 August	2024 June
Origin of data	Italy	Netherlands	Multinational	Multinational	United States
Patient inclusion	Prospective	Prospective	Prospective	Retrospective	Prospective
Age (years), mean ± SD	54 ± 11	66 ± 9	36.1 ± 3.9	57.7 ± 44.5	60.9 ± 9.4
Female, *n* (%)	100 (100%)	415 (36%)	956 (100%)	926 (40%)	304 (29.5%)
BMI (kg/m^2^), mean ± SD	24.9 ± 4.8	26.5 ± 4.0	24.4 ± 5.3	25.2 ± 4.8	24.6 ± 4.4
Cancer type	Breast	Colorectal	Breast	NSCLC (non–small cell lung cancer)	NSCLC (non–small cell lung cancer)
Stable (< 5% weight loss or gain), *n* (%)	98 (58.0%)	184 (65.7%)	370 (53.9%)	—	—
Weight gain > 5%, *n* (%)	32 (18.9%)	16 (5.7%)	227 (33.0%)	421 (18.3%)	120 (11.7%)
Weight loss > 5%, *n* (%)	39 (23.1%)	80 (28.6%)	90(13.1%)	—	—

In this context, Roeland et al. should be congratulated for addressing this important question. In this post hoc analysis, the authors defined different percentage cut‐off values for maximum weight gain among patients with non–small cell lung cancer within 3 months of starting platinum‐based chemotherapy. Among all categories, namely, weight gain > 0%, > 2.5% and > 5%, a significant benefit in overall and progression‐free survival was seen and was comparable among all groups, with the study displaying adequate power. These findings highlight the clinical significance of incorporating strategies that encourage weight gain and prevent weight loss at the least among cancer patients. Furthermore, defining three different cut‐off points can be beneficial for future clinical trials among cancer cachexia patients, and finding an ideal cut‐off among these can guide future endpoint allocation [15, 17].

Conversely, not all patients with significant weight gain demonstrated a survival benefit, raising the question of the cause behind the weight change. A major contributory factor could be oedema, commonly seen among cancer patients. Recognizing the underlying cause of oedema in cancer patients poses a challenge, as it can be attributed to the progression of the tumour, immobility due to their condition and multimorbidity with conditions such as heart failure, renal impairment, anaemia and musculoskeletal disorders [18]. Additionally, polypharmacy plays a role, with medicines such as calcium channel blockers, nonsteroidal anti‐inflammatory agents, antidepressants, antidiabetics or opioids inducing oedema. Oncological management can be implicated in the development of oedema, with platinum‐based chemotherapy, used primarily in this study, leading to acute and chronic renal toxicity. Fluid retention due to cardiovascular disease from chemotherapy, manifesting as arrhythmias, acute myocardial infarction, hypertension, myocarditis and ultimately heart failure, may also contribute to the incidence of oedema among cancer patients [19]. In future clinical trials based on Roeland et al.'s study design, weight gain attributed to oedema must be identified and analysed separately. Furthermore, standard assessments of weight measurement were not clarified, including the weight of clothes and the time the data were taken along with the level of precision. Owing to a large number of patients from the cohort demonstrating weight loss, their impact on the analysis was substantial, with the negative outcomes seen among this group driving the results, diminishing the significance of the survival benefit seen among patients with weight gain. An analysis comparing patients with a stable weight change of approximately 2.5% compared with groups displaying weight loss or different variations of weight gain would further add value to the data.

Several assessment tools can be utilized to distinguish between healthy weight gain versus changes due to oedema. Among them, bioelectrical impedance analysis (BIA) is a noninvasive and cost‐effective method to determine body composition, including fat mass, fat‐free mass and total body water. Another commonly employed method, dual‐energy X‐ray absorptiometry (DXA), is gaining popularity as it can be used as a superior method to assess weight gain, by distinguishing between increases in BMI that can be attributed to nonfat mass compared with fat mass—potentially useful in the management of oncological management [20]. For patients at risk or diagnosed with heart failure, the Remote Dielectric Sensing System (ReDS) is emerging as one with the potential to detect and monitor pulmonary congestion, thus guiding pharmacological management, and can be used among cancer patients to rule out cardiogenic oedema [21].

In conclusion, weight changes hold significant value as prognostic factors, with weight loss guiding the diagnosis of cancer cachexia and weight gain leading to improved overall and progression‐free survival. Incorporating nutritional and physical rehabilitation that aims to ensure weight preservation and gain among cancer patients can positively impact outcomes. To do so, future studies should thoroughly assess and report weight changes, including their underlying causes along with their impact on tumour burden and survival among cancer patients.

## Conflicts of Interest

MSK reports fees from Bayer and Novartis. JB reports consulting fees from Abbott, American Regent, Amgen, Applied Therapeutics, AskBio, Astellas, AstraZeneca, Bayer, Boehringer Ingelheim, Boston Scientific, Bristol Myers Squibb, Cardiac Dimensions, Cardiocell, Cardior, Cardiorem, CSL Behring, CVRx, Cytokinetics, Daxor, Edwards, Element Science, Faraday, Foundry, G3P, Innolife, Impulse Dynamics, Imbria, Inventiva, Ionis, Lexicon, Lilly, LivaNova, Janssen, Medtronic, Merck, Occlutech, Owkin, Novartis, Novo Nordisk, Pfizer, Pharmacosmos, Pharmain, Prolaio, Regeneron, Renibus, Roche, Salamandra, Sanofi, SC Pharma, Secretome, Sequana, SQ Innovation, Tenex, Tricog, Ultromics, Vifor, and Zoll, as well as honoraria from Novartis, Boehringer Ingelheim–Lilly, AstraZeneca, Impulse Dynamics, and Vifor. MSA has no relationships relevant to the contents of this paper to disclose.
